# Differential Association of Serum TSH with Differentiated Thyroid Cancer Risk by Autoimmune Thyroiditis Status

**DOI:** 10.3390/biomedicines13102451

**Published:** 2025-10-08

**Authors:** Lu Yu, Hanyu Wang, Xiao Chen, Yuhan Zhang, Jiaqi Liu, Yang Chen, Yuxin Yu, Siqi Wang, Yu Wang, Zixuan Wang, Lejing Xie, Hui Sun

**Affiliations:** 1 Department of Endocrinology and Metabolism, Union Hospital, Tongji Medical College, Huazhong University of Science and Technology, Wuhan 430000, China; 2 Hubei Medical Clinical Research Center for Diabetes and Metabolic Diseases, Wuhan 430000, China

**Keywords:** differentiated thyroid cancer, thyroid-stimulating hormone, autoimmune thyroiditis, central thyroid hormone sensitivity, thyroid autoantibodies

## Abstract

**Background**: While elevated thyroid-stimulating hormone (TSH) is a known risk factor for differentiated thyroid cancer (DTC), it remains unclear whether autoimmune thyroiditis (AT) modifies this association. Clarifying this interaction is critical for personalized risk assessment and TSH suppression therapy. **Methods**: This study performed a retrospective analysis including 2425 participants who underwent thyroidectomy for thyroid nodules. Participants were categorized based on histological AT diagnosis and thyroid peroxidase antibody (TPOAb) and thyroglobulin antibody (TgAb) levels. Multivariable logistic regression models were used to assess the association between thyroid parameters and DTC risk, stratified by AT and autoantibody status. **Results**: The prevalence of histological diagnosed AT, TgAb-positivity, and TPOAb-positivity among DTC patients was 31.58%, 13.68%, and 18.76%, respectively. An increase in one standard deviation in TSH, thyrotrophic thyroxine resistance index (TT4RI), and TSH index (TSHI) was associated with an elevated risk of DTC in euthyroid individuals without AT or positive thyroid autoantibodies. A positive and nonlinear association between TSH and DTC risk in euthyroid patients without AT was identified, with inflection points at TSH levels of 1.32 mIU/L. In subgroups characterized by concurrent AT, TgAb-positivity, or TPOAb-positivity, these thyroid parameters showed no statistically significant correlation with DTC risk. **Conclusions**: The association between TSH and DTC risk varies according to autoimmune thyroiditis status. These findings highlight the importance of considering thyroid autoimmunity in thyroid cancer risk assessment and warrant prospective evaluation to determine its potential implications for TSH suppressive therapy.

## 1. Introduction

Thyroid cancer is among the most common endocrine malignancies globally [1,2]. The hypothalamic–pituitary–thyroid (HPT) axis plays a fundamental role in thyroid cancer biology, influencing disease development, clinical management, and long-term outcomes [1,3]. Current clinical guidelines recommend thyroid-stimulating hormone (TSH) suppression therapy using supraphysiological levothyroxine doses for patients with intermediate- to high-risk thyroid cancer [4]. Autoimmune thyroiditis (AT), alternatively known as chronic lymphocytic thyroiditis, is the primary cause of acquired hypothyroidism in adults [5], and frequently coexists with thyroid cancer [6]. A meta-analysis indicates that approximately 27.3% of patients with papillary thyroid carcinoma (PTC) also have concurrent AT [7]. Emerging evidence suggests that thyroid autoimmunity may significantly impact the incidence, pathological characteristics and prognosis of differentiated thyroid cancer (DTC) [6,7,8,9,10,11,12,13,14,15], with most studies suggesting a protective effect against aggressive pathological features and disease recurrence [6,7,12,13]. However, the precise relationship between the HPT axis and the development and progression of DTC, particularly in the context of thyroid autoimmunity, remains inadequately understood [3].

The relationship between TSH and thyroid cancer development in patients with AT remains an area characterized by limited and conflicting evidence [16,17,18,19]. A comprehensive meta-analysis demonstrated that higher serum TSH concentrations are associated with increased thyroid cancer risk, with thyroid autoimmunity potentially partially elucidating this association [16]. A recent study utilizing data from the National Health and Nutrition Examination Survey (NHANES) identified a positive correlation between TSH levels and PTC risk, independent of AT [18]. Conversely, Paparodis et al. found no significant association between normal-range TSH concentrations and DTC risk in cases of histologically confirmed AT [17]. The reason for the inconsistency is likely attributable to different criteria for inclusion of the population, differences in sample size, and methodological inconsistencies. The diagnostic criteria for AT have been inconsistent across studies, with some relying solely on histological findings [17] while others depending exclusively on thyroid peroxidase antibody (TPOAb) or thyroglobulin antibody (TgAb) levels [18]. Moreover, the histological diagnostic standards for AT exhibit considerable regional variability, potentially leading to misclassification or underdiagnosis of thyroid autoimmune conditions. Notably, the association between DTC risk and TSH levels has not been sufficiently investigated across different thyroid autoantibody profiles, including the TgAb-positive/negative and TPOAb-positive/negative subgroups.

The HPT axis functions via complex negative feedback loop, wherein thyroid hormones negatively regulate TSH secretion [20]. Recently, several studies employed thyroid hormone sensitivity indices to comprehensively evaluate thyroid status [21,22,23,24,25]. Central thyroid hormone sensitivity parameters, including the thyroid feedback quantile-based index (TFQI) [21], TSH index (TSHI) [26], and thyrotrophic thyroxine resistance index (TT4RI) [27], are indicative of the status of the negative feedback loop [21]. Impaired central thyroid hormone sensitivity results in diminished pituitary responsiveness to fluctuations in thyroid hormone levels [24]. Several recent studies have found that impaired central thyroid hormone sensitivity was associated with diabetes [21,24], metabolic syndrome [21], hyperuricemia [22,23], and decreased vitamin D levels [25]. A recent study discovered that reduced central sensitivity to thyroid hormones is linked to increased PTC risk in euthyroid subjects [28]. Notably, further research is needed to consider the potential confounding effect of thyroid autoimmunity on these associations, and to validate the generalizability of the findings within the broader population, including individuals with both normal and abnormal thyroid function.

To derive more dependable insights into this contentious issue, our study aimed to: (1) investigate the association between TSH, central thyroid hormone sensitivity indices, and DTC risk in both euthyroid and general populations with thyroid nodules; and (2) systematically evaluate these relationships across different thyroid autoimmune statuses, specifically within histologically confirmed AT and non-AT subgroups, as well as among populations stratified by TgAb and TPOAb positivity or negativity.

## 2. Methods

### 2.1. Participants

**Figure 1:**
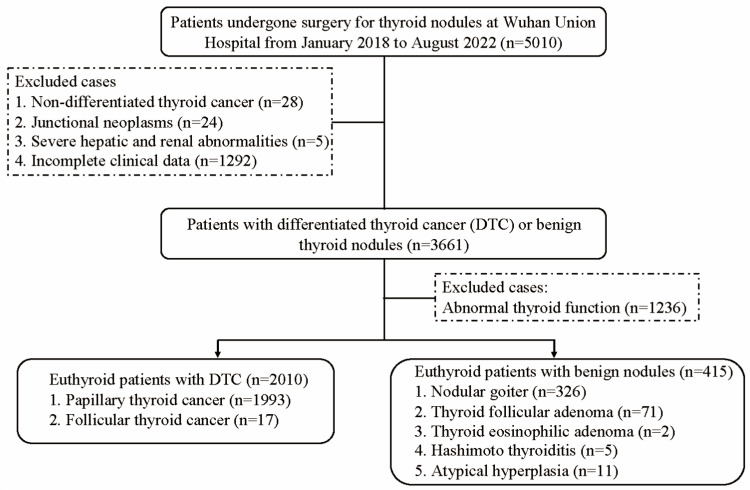
Flowchart of participant recruitment.

This study initially enrolled 5010 patients who underwent thyroidectomy for thyroid nodules at the Wuhan Union Hospital between January 2018 and August 2022 (Figure 1). The exclusion criteria were as follows: (1) non-DTC thyroid cancer, (2) junctional neoplasms, (3) severe hepatic and renal abnormalities, and (4) incomplete clinical data. Ultimately, the study cohort comprised 3661 participants for the sensitivity analysis. After excluding individuals with abnormal thyroid function, the final euthyroid cohort for the main analysis consisted of 2425 eligible participants, including 2010 DTC patients and 415 benign thyroid nodule controls. This study was performed in compliance with the Declaration of Helsinki (2013 revision) and was conducted by the Institutional Review Board of Wuhan Union Hospital. Written informed consent was obtained from all the participants.

### 2.2. Data Collection

Basic information was collected from the participants, including sex, age, height, and weight. Body mass index (BMI) was calculated as weight (kg)/height (m)^2^. Preoperative TSH, free thyroxine (FT4), free triiodothyronine (FT3), TgAb, and TPOAb levels within one month prior to surgery were quantified using a supersensitive electrochemiluminescence immunoassay on a Siemens Centaur XP system. The established reference intervals were: TSH (0.27–4.2 mIU/L), FT4 (12–22 pmol/L), FT3 (3.1–6.8 pmol/L), TgAb (<115.0 IU/mL), and TPOAb (<34.0 IU/mL).

### 2.3. Assessment of Thyroid Autoimmune Status

The histological diagnosis of AT [12] was established based on comprehensive evaluation of surgical specimens, requiring the presence of all following features in non-neoplastic thyroid tissue: (1) diffuse lymphoplasmacytic infiltration with formation of lymphoid follicles containing germinal centers; (2) oncocytic (Hurthle cell) metaplasia of follicular epithelium; (3) variable degrees of follicular atrophy and interstitial fibrosis. These characteristic changes had to be clearly distinct from tumor-associated inflammatory responses and present in areas remote from the primary tumor site. TgAb-positivity was defined as TgAb > 115.0 IU/mL. TPOAb-positivity was defined as TPOAb > 34.0 IU/mL.

### 2.4. Central Thyroid Hormone Sensitivity Indices

TT4RI = FT4 (pmol/L) × TSH (mIU/L) [27]. TSHI = Ln TSH (mIU/L) + 0.1345 × FT4 (pmol/L) [26]. TFQI = empirical cumulative distribution function (cdf) FT4 − (1 − cdf TSH) [21]. The TFQI values span from −1 to 1. Higher positive values of TFQI, TSHI, and TT4RI indicate reduced central sensitivity to thyroid hormones.

### 2.5. Statistical Analysis

The correlations between thyroid parameters (TSH, TT4RI, TSHI and TFQI) and DTC risk were evaluated utilizing multivariable logistic regression models. When used as a categorical variable, the thyroid parameters were divided into tertiles. Age, sex, and BMI were modified in the adjusted model. To account for potential effect modification by thyroid autoimmunity, we performed stratified analyses based on autoimmune status, categorizing participants into: (1) AT versus non-AT; (2) TPOAb-positive versus TPOAb-negative; and (3) TgAb-positive versus TgAb-negative groups. We employed smoothing techniques and generalized additive models to investigate the potential nonlinear relationships between thyroid parameters and DTC risk.

To verify the robustness of the findings, sensitivity analyses were performed on the overall cohort (*n* = 3661), encompassing participants with normal and abnormal thyroid function. In the overall cohort, TSH levels were categorized as follows: TSH < 0.27 mIU/L, TSH ≥ 4.2 mIU/L, and 0.27 ≤ TSH < 4.2 mIU/L. Participants within the 0.27 ≤ TSH < 4.2 mIU/L range were further stratified into three groups based on tertile distribution.

Statistical analyses were conducted using Empower Stats (http://www.empowerstats.com) and R package (https://www.r-project.org/). *p* < 0.05 (two-sided) was employed to determine statistical significance.

## 3. Results

### 3.1. Characteristics of Participants

Baseline characteristics of 2425 participants diagnosed with DTC or benign thyroid nodules are presented in Table 1. The mean age of the participants was 47.21 ± 10.39 years, with 1840 (75.85%) being females. A sum of 2010 (82.89%) was diagnosed with DTC. The participants with DTC exhibited a higher likelihood of being younger, TgAb-positive, TPOAb-positive, and having a combination of histologic diagnosis of AT, compared to those with benign nodules. They also had higher levels of TSH, TT4RI, TSHI, and TFQI levels (all *p* < 0.05). The prevalence of histological diagnosed AT, TgAb-positivity, and TPOAb-positivity among DTC patients was 31.58%, 13.68%, and 18.76%, respectively.

### 3.2. The Relationship Between TSH and Central Thyroid Hormone Sensitivity with the Risk of DTC in a Population with Normal Thyroid Function

As shown in Table 2, an increase in one standard deviation (SD) in TSH, TT4RI, and TSHI was associated with an elevated risk of DTC. The adjusted odds ratios (ORs) were calculated as 1.27 (95% CI: 1.14–1.43, *p* < 0.001) for TSH, 1.24 (95% CI: 1.11–1.39, *p* < 0.001) for TT4RI, and 1.20 (95% CI: 1.08–1.33, *p* = 0.001) for TSHI, after controlling for age, sex, and BMI. Additionally, when comparing the highest tertiles to the lowest tertiles, the risk of DTC increased by 59% for TSH (OR = 1.59, 95% CI: 1.23–2.07, *p* < 0.001), 50% for TT4RI (OR = 1.50, 95% CI: 1.16–1.96, *p* = 0.002), and 32% for TSHI (OR = 1.32, 95% CI: 1.01–1.71, *p* = 0.039), with all *p*-values for trend being less than 0.05.

### 3.3. Stratified Analysis

**Figure 2:**
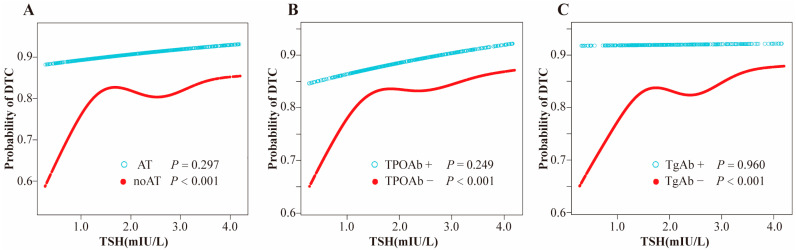
(**A**) Smooth curve fitting model for nonlinear relationship of TSH with DTC stratified by AT; (**B**) Smooth curve fitting model for nonlinear relationship of TSH with DTC stratified by TPOAb; (**C**) Smooth curve fitting model for nonlinear relationship of TSH with DTC stratified by TgAb. Adjusted for age, sex and body mass index. Abbreviations: TSH, thyroid-stimulating hormone; DTC, differentiated thyroid cancer; AT, autoimmune thyroiditis; TPOAb, thyroid peroxidase antibody; TgAb, thyroglobulin antibody.

Stratified analyses were performed to explore the association between thyroid parameters (TSH, TT4RI, TSHI, and TFQI) and DTC risk across different thyroid autoimmune statuses, including AT, TPOAb, and TgAb statuses (Figure 2). The results revealed significant positive associations between higher TSH, TT4RI, and TSHI levels and increased DTC risk, exclusively in individuals without thyroid autoimmunity. The adjusted odds ratios (ORs) were calculated as 1.25 (95% CI: 1.10–1.43, *p* < 0.001) for TSH, 1.22 (95% CI: 1.06–1.39, *p* = 0.004) for TT4RI, and 1.18 (95% CI: 1.08–1.34, *p* = 0.007) for TSHI in participants without histologic diagnosed AT, after controlling for age, sex, and BMI. However, in subgroups characterized by concurrent AT, TgAb-positivity, or TPOAb-positivity, these thyroid parameters showed no statistically significant correlation with DTC risk. This phenomenon was also observed in the relationship between tertiles of TSH and TT4RI and DTC risk.

### 3.4. The Nonlinear Relationship Between TSH and DTC Risk in Participants Without Thyroid Autoimmunity

**Figure 3:**
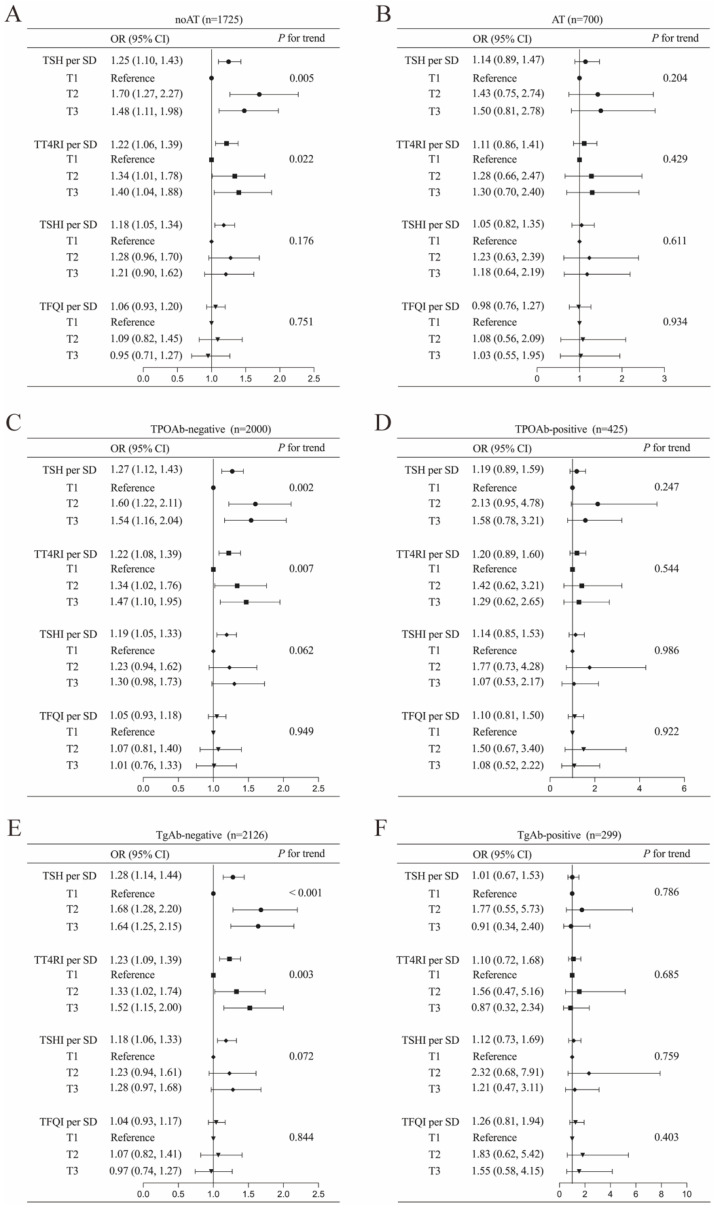
Associations of TSH and central thyroid hormone sensitivity with DTC in a euthyroid population stratified into the following subgroups: (**A**) autoimmune thyroiditis (AT), (**B**) non-autoimmune thyroiditis (noAT), (**C**) thyroid peroxidase antibody (TPOAb)-negative, (**D**) TPOAb-positive, (**E**) thyroglobulin antibody (TgAb)-negative, and (**F**) TgAb-positive. Adjusted for age, sex and body mass index. Abbreviations: TSH, thyroid-stimulating hormone; DTC, differentiated thyroid cancer; OR, odds ratio; CI, confidence interval; SD, standard deviation; TT4RI, thyrotrophic thyroxine resistance index; TSHI, TSH index; TFQI, thyroid feedback quantile-based index.

Utilizing a model with smooth curve fitting, a positive, nonlinear association between TSH and DTC risk was identified in euthyroid participants without thyroid autoimmunity, with all *p*-values < 0.05 (Figure 3). The inflection points for TSH were calculated at 1.32, 1.32, and 1.41 in subjects with non-AT, TPOAb-negative or TgAb-negative, respectively, as determined by a binary linear regression model and recursive algorithm (log-likelihood ratio test *p* < 0.05).

### 3.5. Sensitivity Analysis

To validate the robustness of our findings, we extended our investigation to a broader population encompassing individuals with both normal and abnormal thyroid function. This sensitivity analysis yielded patterns consistent with our primary results. Higher levels of thyroid indices (TSH, TT4RI, TSHI, and TFQI) demonstrated significant associations with heightened DTC risk, after controlling for age, sex, and BMI (Table 3). Notably, within the normal TSH range, higher TSH tertiles were linked to an elevated DTC risk. However, no significant associations were identified between DTC risk and TSH levels below or above the normal range when compared to the first tertile of normal-range TSH.

**Figure 4:**
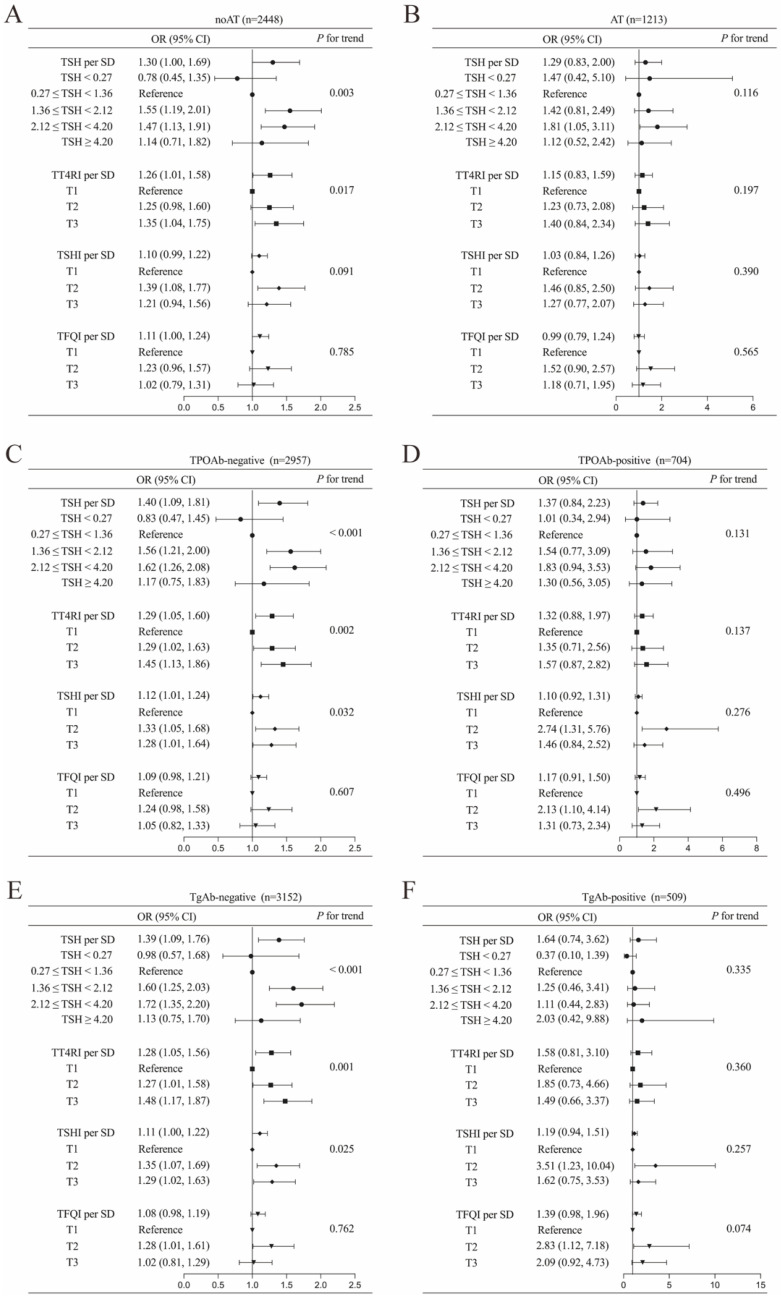
Associations of TSH and central thyroid hormone sensitivity with DTC in overall population stratified into the following subgroups: (**A**) autoimmune thyroiditis (AT), (**B**) non-autoimmune thyroiditis (noAT), (**C**) thyroid peroxidase antibody (TPOAb)-negative, (**D**) TPOAb-positive, (**E**) thyroglobulin antibody (TgAb)-negative, and (**F**) TgAb-positive. Adjusted for age, sex and BMI. Abbreviations: TSH, thyroid-stimulating hormone; DTC, differentiated thyroid cancer; OR, odds ratio; CI, confidence interval; SD, standard deviation; TT4RI, thyrotrophic thyroxine resistance index; TSHI, TSH index; TFQI, thyroid feedback quantile-based index.

Subgroup analyses in this expanded cohort revealed that the positive correlations between thyroid parameters and DTC risk were particularly pronounced in individuals without evidence of thyroid autoimmunity (all *p* < 0.05). Conversely, among participants with concurrent AT and positive thyroid autoantibodies (TgAb and TPOAb), no statistically significant correlation was observed (Figure 4).

## 4. Discussion

This study comprehensively investigated the association between TSH, central thyroid hormone sensitivity, and the risk of DTC in a large cohort of patients with thyroid nodules across diverse thyroid autoimmune statuses. Our findings revealed that higher normal-range TSH levels and impaired central thyroid hormone sensitivity were significantly associated with increased DTC risk, but only in individuals with the absence of thyroid autoimmunity. In contrast, no such association was observed in patients with histologically confirmed AT or positive thyroid autoantibodies.

TSH exerts its biological effects through binding to TSH receptors (TSHR) on thyroid follicular cells, regulating cellular proliferation and differentiation [29]. Preclinical evidence indicates that TSH may promote tumor progression, including proliferation, invasion, and immune evasion, particularly in thyroid cancer models exhibiting high TSHR expression [30]. However, the responsiveness of thyroid tumors to TSH stimulation demonstrates substantial heterogeneity, likely attributable to variations in TSHR expression levels and functionality [3]. Notably, TSHR expression exhibits an inverse correlation with the differentiation status of thyroid tumors [3]. Current evidence indicates that while TSH-TSHR signaling promotes the proliferation of thyroid follicular cells, it alone is insufficient to initiate carcinogenesis [3]. The development of aggressive thyroid cancer phenotypes appears to require other coexisting mutations that activate complementary signaling pathways [3,31].

AT is characterized by an enhanced immune response against the thyroid gland which can ultimately result in reduced thyroid hormone synthesis [32]. It is the most prevalent cause of hypothyroidism in adults [5,32]. Furthermore, thyroid autoimmune diseases frequently coexist with thyroid cancer [6]. A meta-analysis encompassing 39 studies with a total population of 28,143, found that the prevalence of PTC coexisting with AT was 27.3% [7]. In this study, the prevalence of histological diagnosed AT, TgAb-positivity, and TPOAb-positivity in DTC was 31.58%, 13.68%, and 18.76%, respectively, which are consistent with previous findings [7].

Numerous studies have indicated that patients with PTC coexisting with chronic AT tend to exhibit a less aggressive clinical presentation [6,7,33] and demonstrate improved prognostic outcomes, such as extended recurrence-free survival [13] and decreased PTC-related mortality [12], compared to those with PTC without AT. This may be attributed to the potential influence of thyroid autoimmune on the immune microenvironment of thyroid cancer, potentially enhancing anti-tumor activity [6]. However, several studies present findings that contradict these conclusions [8,34]. The intricate relationship between the HPT axis and the development and progression of DTC, particularly within the context of thyroid autoimmunity, remains insufficiently elucidated.

Currently, the literature examining the association between TSH and the occurrence and progression of thyroid cancer in the context of AT is limited and controversial. Feng et al. reported a positive association between elevated TSH levels and PTC risk independent of AT status [18]. However, while this study controlled for thyroid autoantibodies as a confounding factor, it did not specifically investigate the relationship between TSH and PTC in a population with concurrent AT. Additionally, the study’s reliance on self-reported thyroid cancer diagnoses may introduce selection bias. Our findings are consistent with those of Rodis D. Paparodis et al., which included 2060 participants (1731 without AT and 329 with histologically confirmed AT) after excluding individuals with a history of thyroid dysfunction or abnormal TSH levels [17]. This study revealed that higher TSH levels were associated with a higher incidence of DTC only in the non-AT subgroup, while no significant differences in TSH levels were observed between DTC and non-DTC cases among subjects with AT [17]. Nonetheless, AT can result in elevated TSH levels or even clinical hypothyroidism [5], and the exclusion of participants with abnormal TSH levels in the study by Rodis D. Paparodis et al. may limit the generalizability of their findings. Meanwhile, the absence of standardized histological diagnostic criteria for AT across different regions may introduce variability in the study outcomes. To address these methodological constraints, our study incorporated several key improvements in both study design and analysis. We established a larger cohort with 700 AT cases in the euthyroid cohort and 1213 AT cases in the overall cohort, thereby enhancing the statistical power of our analysis. This analytical approach incorporated multiple stratification criteria, including histological AT diagnosis and thyroid autoantibody profiles (TgAb and TPOAb status). Our study revealed that TSH levels showed no significant association with DTC risk in individuals with concurrent either histologically confirmed AT or positive thyroid autoantibodies (TgAb or TPOAb). Furthermore, we extended our investigation to a broader population encompassing individuals with both normal and abnormal thyroid function, demonstrating consistent results that reinforce the robustness of our analytical framework. To the best of our knowledge, this study constitutes the most comprehensive investigation to date, examining the relationship between TSH and central thyroid hormone sensitivity and DTC risk across varying statuses of thyroid autoimmunity.

The hypothalamic–pituitary axis is suppressed by thyroid hormones, establishing a negative feedback loop [20]. The diminished role of thyroid hormone in the pituitary gland, characterized by reduced sensitivity, implies that TSH secretion becomes less responsive to the modulatory effects of thyroid hormone fluctuations [24]. Several recent studies have found that impaired central thyroid hormone sensitivity was associated with diabetes [24], hyperuricemia [22], and cardiovascular risk [35]. D. Muhanhali et al. found that impaired central sensitivity to thyroid hormones increased the risk of developing PTC in euthyroid subjects [28]. However, their analysis did not account for the potential influence of thyroid autoimmunity. Our findings extend this observation by revealing that the association between impaired central thyroid hormones sensitivity and PTC risk is restricted to individuals without concurrent AT, while no significant association was observed in AT patients. Furthermore, the sensitivity analysis validated the generalizability of the findings.

The precise mechanisms underlying these findings warrant further investigation. In thyroid carcinogenesis, the activation of the mitogen-activated protein kinase pathway leads to the downregulation of TSHR expression and inhibition of adenylate cyclase activity, as mediated by receptor tyrosine kinase (RTK) fusions, RAS or BRAF mutations [29,36,37]. This molecular cascade may impair thyroid hormone biosynthesis, subsequently triggering compensatory TSH hypersecretion through negative feedback mechanisms. Some studies suggest that AT increases the risk of developing PTC based on the theory that chronic inflammation is thought to promote tumorigenesis [6,38,39]. Emerging evidence suggests a potential molecular link between AT and thyroid malignancies, characterized by the co-expression of multiple genes such as TSHR, BTB domain and CNC homolog 2 (BACH2), and forkhead box E1 (FOXE1), along with shared somatic mutations [6,40]. One plausible, but speculative, interpretation of our findings is that inflammatory and immune-related pathways active in AT could contribute to thyroid tumor development through mechanisms that are at least partly independent of classical TSH signaling [6,41]. Specialized mechanism studies and longitudinal research are required to elucidate the precise mechanisms involved.

The American Thyroid Association recommends TSH-suppressive therapy for patients with intermediate- to high-risk DTC following total thyroidectomy and radioiodine therapy, with target TSH levels determined through dynamic risk stratification and treatment response [4,42,43]. However, some study demonstrated that TSH suppression does not improve survival outcomes while increasing the risk of skeletal morbidity and cardiovascular complications [44,45]. The role of TSH suppression therapy is being re-evaluated, particularly with respect to its risk–benefit profile across different patient subgroups [3,44]. In our cohort, the association between serum TSH and DTC risk differed according to the presence of autoimmune thyroiditis. These associative observations raise the possibility that the net benefit of TSH suppression may vary by autoimmune status. However, causal or clinical implications cannot be drawn from our retrospective, cross-sectional data. Prospective studies with clinical endpoints are therefore required to assess whether personalized TSH targets based on thyroid autoimmune status could optimize the risk–benefit balance of suppressive therapy.

Several limitations should be acknowledged. First, as the definitive diagnosis of AT was based on histopathology, our study population primarily comprised patients who underwent thyroidectomy. Consequently, those with thyroid nodules characterized as benign on fine-needle aspiration in outpatient settings were less likely to be included, potentially affecting the generalizability of the absolute risk estimates. Nonetheless, this potential bias is unlikely to significantly affect serum TSH levels or AT status determination, and its impact was mitigated by the large sample size. Second, although we adjusted for key demographic variables (age, sex, and BMI) in line with previous studies [17,18], data on certain potential confounding factors, such as iodine nutritional status, medication use, smoking history, comorbidities, and nodule characteristics were lacking. The residual confounding effects attributable to these factors cannot be entirely ruled out, necessitating further investigation in future studies. Third, the number of TgAb-positive patients was relatively small, which may limit the statistical power of subgroup analyses. However, the consistent results across multiple thyroid autoimmune stratification criteria. Finally, owing to the retrospective nature of the analysis, and the absence of longitudinal outcome data such as recurrence or survival, causal inferences cannot be drawn, therefore the results should be interpreted with caution.

In conclusion, our study provides robust evidence that higher normal-range TSH levels and impaired central thyroid hormone sensitivity are associated with an increased risk of DTC in individuals without thyroid autoimmunity. Conversely, no significant association between thyroid parameters and DTC risk was observed in patients with concurrent histologically confirmed AT or positive thyroid autoantibodies (TgAb/TPOAb). These findings may reflect differences in the interplay between autoimmune-related immune processes and thyroid tumor biology. Prospective cohorts with tissue-level mechanistic investigations and longitudinal clinical outcomes are required to validate these observations and to determine any implications for risk stratification or TSH suppression therapy.

## Figures and Tables

**Table 1:** Baseline characteristics of patients with DTC and patients with benign thyroid nodules exhibiting normal thyroid function.

	Overall	DTC	Benign Nodules	*p* Value
N	2425	2010	415	
Age (year)	47.21 ± 10.39	46.50 ± 10.08	50.60 ± 11.12	<0.001
Female, n (%)	1839 (75.84)	1509 (75.07)	330 (79.52)	<0.001
BMI (kg/m^2^)	23.26 ± 3.21	23.26 ± 3.23	23.28 ± 3.10	0.910
FT3 (pmol/L)	4.44 ± 0.63	4.44 ± 0.63	4.46 ± 0.64	0.531
FT4 (pmol/L)	14.31 ± 1.97	14.28 ± 1.96	14.44 ± 2.00	0.132
TSH (mIU/L)	1.71 (1.20, 2.39)	1.74 (1.25, 2.42)	1.52 (1.03, 2.23)	<0.001
TFQI	0.08 ± 0.30	0.09 ± 0.30	0.05 ± 0.32	0.043
TT4RI	24.23 (16.73, 34.17)	24.55 (17.14, 34.59)	22.78 (14.42, 31.64)	<0.001
TSHI	2.44 (2.06, 2.82)	2.45 (2.08, 2.83)	2.39 (1.94, 2.76)	<0.001
TgAb-positive, n (%)	299 (12.33)	275 (13.68)	24 (5.78)	<0.001
TPOAb-positive, n (%)	425 (17.53)	377 (18.76)	48 (11.57)	<0.001
AT, n (%)	700 (28.87)	635 (31.58)	65 (15.70)	<0.001

Data are presented as mean ± standard deviation (SD), median (interquartile range), or n (%). Abbreviations: DTC, differentiated thyroid cancer; BMI, body mass index; FT3, free triiodothyronine; FT4, free thyroxine; TSH, thyroid-stimulating hormone; TFQI, thyroid feedback quantile-based index; TT4RI, thyrotrophic thyroxine resistance index; TSHI, TSH index; TgAb, thyroglobulin antibody; TPOAb, thyroid peroxidase antibody, AT, autoimmune thyroiditis.

**Table 2:** Associations of TSH and central thyroid hormone sensitivity with DTC in a population with normal thyroid function.

	Unadjusted Model		Adjusted Model	
	OR (95% CI)	*p* Value	OR (95% CI)	*p* Value
TSH per SD	1.28 (1.14, 1.43)	<0.001	1.27 (1.14, 1.43)	<0.001
T 1	Reference		Reference	
T 2	1.66 (1.28, 2.14)	<0.001	1.66 (1.28, 2.16)	<0.001
T 3	1.59 (1.24, 2.06)	<0.001	1.59 (1.23, 2.07)	<0.001
*p* for trend	<0.001		<0.001	
TT4RI per SD	1.26 (1.13, 1.41)	<0.001	1.24 (1.11, 1.39)	<0.001
T 1	Reference		Reference	
T 2	1.37 (1.06, 1.76)	0.015	1.35 (1.04, 1.74)	0.023
T 3	1.55 (1.20, 2.01)	<0.001	1.50 (1.16, 1.96)	0.002
*p* for trend	<0.001		0.002	
TSHI per SD	1.23 (1.11, 1.36)	<0.001	1.20 (1.08, 1.33)	0.001
T 1	Reference		Reference	
T 2	1.32 (1.02, 1.70)	0.033	1.26 (0.98, 1.64)	0.077
T 3	1.39 (1.07, 1.79)	0.013	1.32 (1.01, 1.71)	0.039
*p* for trend	0.012		0.036	
TFQI per SD	1.12 (1.00, 1.25)	0.044	1.07 (0.96, 1.20)	0.224
T 1	Reference		Reference	
T 2	1.15 (0.89, 1.48)	0.295	1.12 (0.86, 1.45)	0.404
T 3	1.15 (0.89, 1.49)	0.289	1.04 (0.80, 1.36)	0.750
*p* for trend	0.286		0.736	

The evaluation of the odds ratio (OR) and 95% confidence interval (CI) was conducted using multivariable logistic regression models. Adjusted model: adjusted for sex, age, body mass index. Abbreviations: TSH, thyroid-stimulating hormone; DTC, differentiated thyroid cancer; SD, standard deviation; TT4RI, thyrotrophic thyroxine resistance index; TSHI, TSH index; TFQI, thyroid feedback quantile-based index.

**Table 3:** Associations of TSH and central thyroid hormone sensitivity with DTC in the overall population.

	Unadjusted Model		Adjusted Model	
	OR (95% CI)	*p* Value	OR (95% CI)	*p* Value
TSH per SD	1.46 (1.18, 1.81)	<0.001	1.46 (1.17, 1.83)	0.001
T 1	Reference		Reference	
T 2	1.54 (1.25, 1.89)	<0.001	1.49 (1.20, 1.86)	<0.001
T 3	1.64 (1.33, 2.03)	<0.001	1.67 (1.34, 2.09)	<0.001
*p* for trend	<0.001		<0.001	
0.27 ≤ TSH < 1.36	Reference		Reference	
TSH < 0.27	0.84 (0.54, 1.33)	0.463	0.94 (0.58, 1.53)	
1.36 ≤ TSH < 2.12	1.57 (1.26, 1.97)	<0.001	1.57 (1.24, 1.98)	<0.001
2.12 ≤ TSH < 4.2	1.68 (1.35, 2.10)	<0.001	1.71 (1.35, 2.16)	<0.001
TSH ≥ 4.2	1.30 (0.91. 1.87)	0.154	1.32 (0.89, 1.94)	0.165
*p* for trend	<0.001		<0.001	
TT4RI per SD	1.39 (1.16, 1.66)	<0.001	1.35 (1.12, 1.63)	0.002
T 1	Reference		Reference	
T 2	1.34 (1.09, 1.65)	0.005	1.30 (1.05, 1.62)	0.017
T 3	1.58 (1.28, 1.95)	<0.001	1.54 (1.23, 1.93)	<0.001
*p* for trend	<0.001		<0.001	
TSHI per SD	1.14 (1.06, 1.24)	0.001	1.12 (1.03, 1.22)	0.011
T 1	Reference		Reference	
T 2	1.50 (1.22, 1.85)	<0.001	1.42 (1.14, 1.78)	0.002
T 3	1.45 (1.18, 1.79)	<0.001	1.37 (1.10, 1.71)	0.005
*p* for trend	<0.001		0.004	
TFQI per SD	1.19 (1.09, 1.31)	<0.001	1.12 (1.02, 1.23)	0.022
T 1	Reference		Reference	
T 2	1.36 (1.10, 1.68)	0.004	1.35 (1.08, 1.69)	0.008
T 3	1.27 (1.03, 1.57)	0.023	1.13 (0.90, 1.40)	0.292
*p* for trend	0.020		0.247	

The evaluation of the odds ratio (OR) and 95% confidence interval (CI) was conducted using multivariable logistic regression models. Adjusted model: adjusted for sex, age, body mass index. Abbreviations: TSH, thyroid-stimulating hormone; DTC, differentiated thyroid cancer; SD, standard deviation; TT4RI, thyrotrophic thyroxine resistance index; TSHI, TSH index; TFQI, thyroid feedback quantile-based index.

## Data Availability

Data underlying the findings of this study are available upon request from the corresponding author.

## Abbreviations

HPT, hypothalamic–pituitary–thyroid; TSH, thyroid-stimulating hormone; AT, autoimmune thyroiditis; PTC, papillary thyroid carcinoma; DTC, differentiated thyroid cancer; TPOAb, thyroid peroxidase antibody; TgAb, thyroglobulin antibody; TFQI, thyroid feedback quantile-based index; TSHI, TSH index; TT4RI, thyrotrophic thyroxine resistance index; BMI, body mass index; FT4, free thyroxine; FT3, free triiodothyronine; TSHR; TSH receptors.
